# The Effect of the Röntgen Ray upon Leukæmic States

**Published:** 1905-12-09

**Authors:** 


### Hospital Clinics.

### THE EFFECT OF THE RÖNTGEN RAY UPON LEUKÆMIC STATES.

A very valuable paper upon the above subject has recently appeared under the hands of Drs. Musser and Edsall.[1] Up to the present we must unhappily admit our therapeutic armament against leukæmia has proved singularly inefficient. It is still too early to speak with assurance of the ultimate prospects of its treatment by the $x$-ray, but there remains little doubt that we have in the latter agent a potent moderator of proteid metabolism, and one which, in the case of leukæmia at least, appears capable of exercising a certain, though ill-understood, selection against the morbid excess of lymphatic tissues which marks the disease.

The authors point out that the potentialities of the $x$-ray, for good and ill alike, are only now beginning to be recognised. It was long believed that the harmful effects of the ray were confined to the skin burns which so frequently in its early days marked its application. But extended knowledge is revealing unsuspected properties inherent in the Röntgen ray—for instance, its capacity to induce sterility or nephritis, or, as occasionally happens, fever, vomiting, and prostration. In the case of leukæmia the action of the rays is, as has been said, in the direction of a proteid catabolism especially affecting the hyperplastic lymphoid tissues.

The paper under notice is based upon two cases of leukæmia, of which one appeared to recover completely under the influence of the treatment, while the other (a relapsed case previously bettered by the light treatment) showed no improvement and progressed uninterruptedly to a fatal issue. The details are as follows:—

Case 1. *Fatal Result.*—A plumber aged forty-four came under notice of Dr. Musser on September 15, 1904, with the following history. He had been in good health until March 1904, but since then had been growing progressively more feeble. He had suffered intermittently from diarrhœa, and was sensible of an unusual degree of mental irritability. He presented on admission the typical clinical picture of spleno-myelogenous leukæmia. The spleen was enormously enlarged, measuring 12½ by 13 inches. The blood examination gave the following results:—

| | |
|---|---|
| Hæmoglobin ... | 38 per cent. |
| Red blood cells ... | 4,280,000 per cubic mm. |
| White blood cells ... | 407,500 per cubic mm. |

A differential count of the white cells resulted as follows:—

| | |
|---|---|
| Myelocytes ... ... ... | 49.2 per cent. |
| Polymorphonuclear ... ... | 27.1 ,, |
| Transitional ... ... ... | 21.6 ,, |
| Eosinophile ... ... ... | 1.0 ,, |
| Lymphocytes ... ... ... | .2 ,, |

Treatment by exposure to the $x$-ray was instituted, and was rewarded by a substantial and rapid improvement. By October 13 the leucocyte count had fallen to 138,000, the spleen was very much reduced in size, and the constitutional condition correspondingly ameliorated. The leucocyte count ultimately fell to 35,000, and the spleen became of normal dimensions. The $x$-ray treatment was continued once or twice a week until February, and then intermitted altogether. On March 12, that is to say, after a discontinuance of the treatment for a month, the patient returned in a completely relapsed state. He complained of pain and soreness in his muscles, his spleen had enlarged again, and the leucocyte count had risen to 180,400. The differential count of leucocytes now appeared as follows:—

| | |
|---|---|
| Neutrophile myelocytes ... | 71.5 per cent. |
| Polymorphonuclear cells ... | 18.0 ,, |
| Lymphocytes ... ... ... | 4.0 ,, |
| Basophile cells ... ... ... | 4.0 ,, |
| Eosinophile myelocytes ... | 2.5 ,, |

The patient on this occasion grew rapidly worse in spite of the application of the $x$-rays. The leucocyte count mounted steadily, reaching 496,000 on March 29, two days before his death.

Case 2. *Apparent Cure.*—A woman aged forty-two came under the observation of Dr. Musser on March 20 of this year, giving the following history. About the month of October 1903 she had first begun to notice that she was more easily wearied than usual. She lost flesh, became pale, and herself observed the lump in her flank presented by the enlarged spleen. She improved for a time, but a year later, on the occasion of a trifling operation upon her eyelid, lost a very large quantity of blood, control over the small wound being established only after several hours. She was a good deal weakened by this experience, and from it there dated a rapid enlargement of the spleen with aggravation of her symptoms. Like the other case she presented the appearance typical of myelogenous leukæmia, her spleen crossing the middle line and reaching downwards to within one inch of Poupart's ligament. Her blood examination gave the following results:—

| | |
|---|---|
| Hæmoglobin ... ... | 68 per cent. |
| Red cells ... ... | 3,410,000 per cubic mm. |
| White cells ... ... | 245,000 per cubic mm. |

*Differential Count.*

| | | |
|---|---|---|
| Neutrophile myelocytes ... ... | 45 per cent. | =64 per cent. of myelocytes. |
| Basophile myelocytes ... ... | 16 ,, | |
| Eosinophile myelocytes ... ... | 3 ,, | |
| Polymorphonuclear cells ... ... | 26 ,, | |
| Lymphocytes ... ... ... | 9 ,, | |

The patient was pallid, weak, and thin, and obviously going fast down hill, for during the six days which elapsed between her admission and the commencement of $x$-ray treatment her leucocyte count rose from 245,000 to 304,000. The effect of the treatment was almost magical, as is evident from a consideration of the leucocyte counts appended.

| | | |
|---|---|---|
| March 26 (treatment begun) ... | ... | 304,000 |
| ,, 27 ,, ... | ... | 262,400 |
| ,, 29 ,, ... | ... | 246,400 |
| April 1 ,, ... | ... | 149,600 |
| ,, 5 ,, ... | ... | 80,000 |
| ,, 15 ,, ... | ... | 28,000 |
| May 8 (treatment ended) ... | ... | 9,040 |

The authors conducted some limited observations upon the course of metabolism under the influence of the $x$-rays in the case of leukæmic subjects. In the successful case there was immediate evidence of an increased proteid catabolism, which appeared also in a healthy subject used as a control; in the unsuccessful case, however, there was no response at all—an indication that the action of the rays is not a direct one, but more in the nature of a stimulus requiring an individual response, the direction of the stimulus being towards autolysis.

In drawing attention to the possible application of the $x$-rays to the treatment of obscure nutritional disorders the authors lay particular stress upon the necessity for caution. It is not to be expected, as they point out, that one should be able to double the tissue destruction at any given time without opening up avenues for disaster through the glutting of the excretory mechanisms of the body with a sudden flood of waste products. The sudden and extensive character of this destruction of tissue is invoked by the authors to explain the occurrence of the peculiar toxic symptoms occasionally noted after exposure of the body to $x$-rays.

[1] University of Penna., Med. Bull., Sept. 1905.

